# Justifications of emotional responses to eliciting situations: A narratological approach to the CAD hypothesis

**DOI:** 10.3389/fpsyg.2022.1019485

**Published:** 2022-12-06

**Authors:** Chengfang Song, Xiaosong Rui, Nini Xie

**Affiliations:** ^1^Department of Linguistics, University of International Business and Economics, Beijing, China; ^2^School of Languages and Literature, Harbin Institute of Technology, Weihai, China; ^3^School of Foreign Languages, Northwest University of Politics and Law, Xi'an, China

**Keywords:** CAD hypothesis, justification, narratological approach, correspondence analysis, appraisal theories of emotion

## Abstract

The CAD hypothesis holds that there is mapping between the three moral emotions (contempt, anger and disgust) and the three moral codes of community, autonomy and divinity. Different from previous designs to establish correlations between emotions and eliciting situations which instantiate moral codes, this paper takes a narratological approach to the CAD hypothesis by examining the relationships between the three moral emotions and moral judgment relating to the three moral codes in the context of eliciting situations. First, similarity data pertaining to eliciting situations were collected by using the *Order k/n-1 with fixed K* method. Second, the participants were instructed to write down both their responses and justifications of their responses to the eliciting situations. A narratological analysis of the justifications of responses show that they vary along three variables: narrator, character, and basis (mostly in the form of moral judgment). The descriptive statistics of participants’ responses and of their justifications show that more than a half of responses are in the categories of anger (24.8%), disgust (20.7), and contempt (7.7%) and that about 60% of justifications contain a component of moral judgment based on the three moral codes of autonomy (30.03%), divinity (18.1), and community (11.82%). Correspondence analyses among eliciting situations, emotional responses and the three variables of justifications, together with results from the Multidimensional Scaling analysis of the similarity data, show that the CAD hypothesis is largely supported if mappings are set between the emotions in question and moral judgment concerning the eliciting situations (the basis variable of justification) and that the hypothesis is conditioned by the variable of character.

## Introduction

Based on Shweder et al.’s (1997) distinction of the ethics in community, autonomy, and divinity, Rozin et al. (1999) proposed a CAD triad hypothesis that maps the three moral emotions (contempt, anger, and disgust) and the three moral codes (community, autonomy, and divinity). The community code is based on regulative concepts including “duty, hierarchy, interdependency, and souls,” and aims to protect the moral integrity of the various roles that constitute a community; the autonomy code bases itself on regulative concepts like “harm, rights, and justice,” and aims to protect personal freedom and to cherish the pursuit of personal preferences; and lastly, the divinity code relies on regulative concepts such as “sacred order, natural order, tradition, sanction, sin, and pollution,” with the aim to “protect the soul, the spirit, the spiritual aspects of the human agent and ‘nature’ from degradation” (Shweder et al., 1997, 138). The CAD hypothesis adopts an isomorphic framework (Gray et al., 2017) to set correspondences between specific emotions and types of moral violations. Rozin et al.’s (1999) original design first asked study participants to match 46 situations designed to instantiate the three moral violations to facial illustrations of contempt, anger, and disgust in Task 1, and to verbal expressions of the same set of emotions in Task 2. The preliminary findings based on the two matching tasks were then supported by a classification task that instructed another group of study participants to classify the situations in relation to the three moral violations. Finally, Rozin et al. (1999) confirmed their results by showing that the facial expressions actually made by study participants to the same set of situations were indeed those of contempt, anger, and disgust.

This hypothesis has attracted much attention from researchers. Some of them provided support to the one-to-one correlations between contempt and the moral code of community, between anger and the autonomy code, and between disgust and the divinity code (for example, Li et al., 2016; Dastani and Pankov, 2017). However, the CAD hypothesis has also received critical responses based on a variety of studies. Firstly, the correspondences between the three moral emotions and the three moral codes are not neatly aligned as claimed. For example, the emotion of anger was frequently reported in situations involving the moral violations both in the domain of autonomy and in the domain of community (Russell et al., 2013; Kollareth and Russell, 2017; Kollareth et al., 2019), and the emotion of disgust failed to be recorded as a response to any type of moral violation (Piazza and Landy, 2020). Secondly, some parameters abstracted from different types of moral violations, rather than the categorical moral violations themselves, were found to provide a better account of the elicitation of different emotional responses. One of them is the distinction between act- and character-orientation that applies to all situations, and this basic distinction, according to Giner-Sorolla and Chapman (2017), played the differential role in the elicitation of either anger or disgust. Furthermore, Molho et al. (2017) contended that changes of costs imposed by moral violations differentially triggered anger or disgust, which, in turn, resulted in distinct aggressive strategies toward norm violators. Hartsough et al. (2020) showed that the distinction between second-party and third-party norm violations accounted for the elicitation of either anger or outrage. Thirdly, it was contended that correlation between the emotion of disgust and divinity violations was in need of further elaboration. The emotion was more specifically related to sex- and pathogen-related divinity violations, but was neither sensitive to the situation of child abuse (Kollareth and Russell, 2017) nor to that of self-harm which was not coupled with pathogens (Kollareth and Russell, 2018).

However, it is too early to reject the CAD hypothesis on the basis of the findings reported above. The different findings may result from the different designs they employed. Their designs differed from Rozin et al. (1999) in three different ways. First, the eliciting situations were different in numbers, ranging from 1 (Piazza and Landy, 2020) to 156 (Li et al., 2016), and they were controlled in different degrees, with some having the same story frame changing with one variable (for example, Giner-Sorolla and Chapman, 2017), while others were verbal texts with diverse details. Furthermore, the elicitors were extended from made-up verbal vignettes to include real-life experiences (Molho et al., 2017) and economic games (Hartsough et al., 2020). Second, the fixed set of emotion terms employed in Rozin et al. (1999) was partially replaced (Piazza and Landy, 2020) or enlarged (Kollareth and Russell, 2017). The most important difference comes from the different degrees of emphasis put on the moral judgments of eliciting situations. In Rozin et al. (1999), moral judgment of the situations was made in the classification task and was not treated as an independent variable. Moral judgment received more attention in Kollareth and Russell (2017) and Kollareth et al. (2019), which instructed study participants to rate the immorality of the behavior of the perpetrator in the eliciting situation using a 7-point Likert scale. More significantly, moral judgment was treated as a key variable in the investigation of judgment-emotion correlations in Giner-Sorolla and Chapman (2017), Molho et al. (2017), and Piazza and Landy (2020).

The third difference, in fact, introduces moral judgment as a third element into the CAD hypothesis, which reflects new developments in emotion studies. The schematic account of emotional episodes used to hold that emotions were elicited by events and led to thought/action responses (White, 2000), which possibly motivated the research effort to establish direct relationships between emotions and eliciting situations as found in Rozin et al. (1999). However, more recent appraisal theories of emotion contend that there is an appraisal component between eliciting situations and emotions (Moors et al., 2013). Moreover, Moors et al. (2013) argue that “there is a variable relation between stimuli and emotions, but a stable relation between appraisals and emotions” in that “the same appraisals lead to the same emotions; different appraisals lead to different emotions.” The appraisal component is evaluative in nature and includes the moral judgment mentioned above. The close relation between emotion and appraisal as advocated by the appraisal theories of emotion finds support in Sznycer and Lukaszewski (2019) who identify that the five social emotions form a separate stable constellation with different types of evaluation.

In light of the appraisal theories of emotion, the data collection instructing study participants to match situations to a fixed set of emotions possibly neglected the variable relationships between situations and their moral judgment by treating one as the instantiation of the other. Kollareth and Russell (2017) noted this weakness and indicated that more studies were needed to investigate the “reasons people have for choosing the emotion they do.” The possible variable relationships between eliciting situations and moral judgment undermine the challenges from the studies reviewed above for two reasons. Firstly, the studies that reported conflicting findings possibly did not focus on the same sets of moral emotions and moral codes because their situations may not exemplify the moral codes as expected. Secondly, their data collection using the forced-choice method was highly prone to the false dilemma fallacy because emotional responses might be diverse due to unexpected appraisals to the eliciting situations and were not adequately lexicalized by their fixed sets of emotion words.

In sum, the CAD hypothesis is designed to map the relationships between moral emotions and moral codes; however, the original design and other related research has largely focused on the relationships between moral emotions and so-called instantiations of moral codes, that is, eliciting situations. The appraisal theories of emotion suggest that the equation of moral codes with eliciting situations might be problematic and that it would be better to relate moral codes to moral judgment of eliciting situations (Moors et al., 2013). This paper aims to examine the CAD hypothesis from the perspective of the appraisal theories of emotion by investigating the relationships between the three moral emotions (contempt, anger, and disgust) and moral judgment relating to the three moral codes (community, autonomy, and divinity) in the context of eliciting situations. While fully aware of the possible false dilemma fallacy, a narratological approach is taken to collect open-ended text data. The narratological approach examines the CAD hypothesis through answering the following three questions:

1. What emotional responses do study participants report to the eliciting situations used in Kollareth and Russell (2017)? Specifically, are the responses classifiable into the three condemning social emotions (contempt, anger, and disgust)?
2. How do study participants justify their emotional responses? Are their justifications classifiable in terms of the three moral codes (autonomy, community, and divinity)?
3. Are there any patterns among situations, elements of justifications, and emotions? If yes, how do they relate to the CAD hypothesis?

## The narratological approach and its concepts to be used

De Sousa (2013) has highlighted the importance of narrative in emotion studies. In addition to allowing us to collect open-ended responses, the narratological approach also provides us with a much richer toolkit to analyze emotional episodes in terms of narrative structure, character, the narrator, and the interpersonal resources that construe attitude (including emotion) and establish writer-reader relationships.

Based on linguistic analyses of authentic texts, Hoey (1983, 51) abstracted a basic discourse structure composed of the following elements: situation (which presents the setting), aspect of situation requiring a response (usually a problem in the setting), response (to the problem), evaluation (of the problem), and basis (of evaluation). An approximate correspondence can be set between the textual elements and the components of emotional episodes as proposed in the appraisal theories of emotion: the first two elements in Hoey’s (1983, 53) discourse structure, that is, situation and aspect of situation requiring a response, to the component of the eliciting situation, the middle element of response to the component of emotions or emotional responses, and the last two elements of evaluation and the possible basis in Hoey’s (1983, 53) to the component of appraisal in an emotional episode. Martin and Rose (2008) give more elaborate classifications of situation and of emotional responses and evaluation as well. According to them, the situation can be classified into five categories: record, remarkable event, incident, event description, and complication (Martin and Rose, 2008, 52).

**Figure 1:**
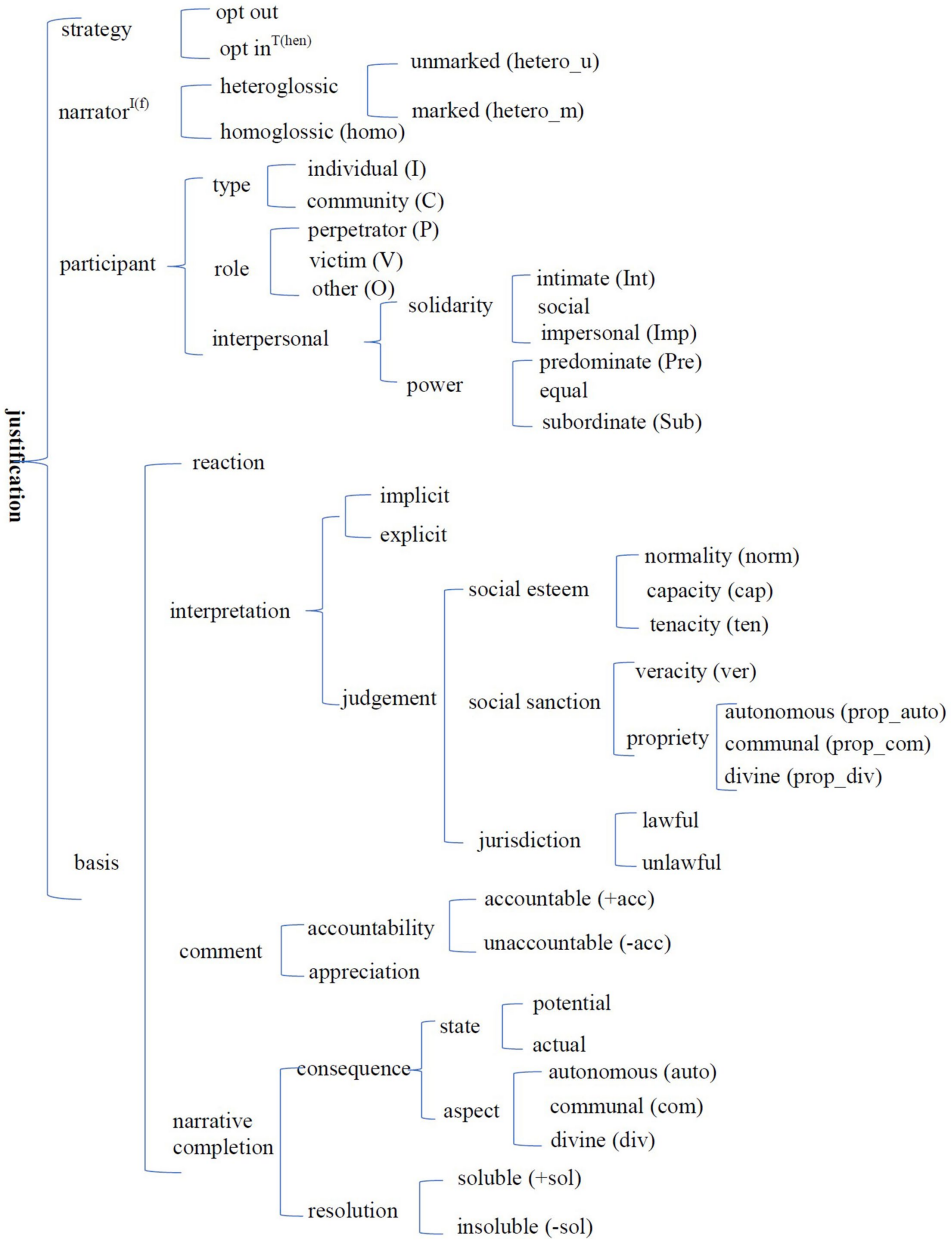
System network of justification.

Martin and White (2005, 42–58) construct all emotional and evaluative resources into a system network of attitudes, which consists of three subcategories: affect, judgment, and appreciation. Affect is concerned with “registering positive and negative feelings,” judgment deals with “attitudes towards behaviour,” and appreciation involves “evaluations of semiotic and natural phenomena, according to the ways in which there are valued or not in a given field” (Martin and White, 2005, 42–43). The three subcategories are divided into positive and negative along the dimension of value, and classified into more elaborate categories according to their contents which are summarized and exemplified in Tables 1–4. The three moral emotions in the CAD hypothesis can all find a place in the system: anger is a case of displeasure (annotated as –sat|disp in the paper), disgust is a case of antipathy (coded as –hap|anti), and contempt is a combination of negative satisfaction and negative judgment (coded as –sat|o-JUDG).^1^ The subcategory of propriety in Table 3, in fact, is closely related to the three moral codes (see Figure 1). Moreover, attitude has the feature of gradability, which means that each attitudinal item is either median, up-scaled, or down-scaled (Martin and White, 2005, 136).

**Table 1:** Categories of affect^1^.

AFFECT	Positive (+)	Negative (−)
desire (des)	long for	(44–1) fearful (*hàipà*)
happiness (hap)	Cheer: cheerful	Misery: (41–4) sad (*shāngxīn*)
	Affection: love	Antipathy: (27–1) abhor (*tònghèn*) | (37–2) vomiting (*fǎnwèi*)
security (sec)	Confidence: assured	Disquiet: (59–3) uneasy (*bùān*) | (1–6) caution (*jǐngtì*)
	Trust: comfortable with	Surprise: (30–3) startled (*jīnghuāng*)
satisfaction (sat)	Interest: (17–2) curious (*haoqi*)	Ennui: (46–3) jaded (*mòrán*) | (46–2) tune out (*shì bù guānjǐ, gāogāo guàqǐ*)
	Pleasure: satisfied	Displeasure: (28–3) angry (*shēngqì*)

1 The emotion words that this paper collects are mostly negative. The examples in the positive column of the table and the following tables that are not followed by a bracket for citation are from Martin and White (2005).

**Table 2:** Categories of judgment of esteem.

SOCIAL ESTEEM	Positive [admire] (+)	Negative [criticize] (−)
normality (norm)	(51–2) normal (*zhèngcháng de*)	(11–4) quirk (*guàipì*)
capacity (cap)	(42–9) civilized (*wénmíng*)	(3–1) stupid (*yúchǔn*)
tenacity (ten)	(56–1) sense of responsibility (*zérèn gǎn*)	(4–4) cowardly (*nuòruò*)

**Table 3:** Categories of judgment of sanction.

SOCIAL SANCTION	Positive [praise] (+)	Negative [condemn] (−)
veracity (ver)	(9–5) credibility (*chéngxìn*)	(6–5) deceitful (*nòngxūzuòjiǎ*)
propriety (prop)	(46–4) kind-hearted (*shànliáng*)	(4–1) selfish (*zìsī*)

**Table 4:** Categories of appreciation^1^.

APPRECIATION	Positive (+)	Negative (−)
reaction (reac)	(36–4) pure and angelic (*tiānzhēnwúxié*)	(16–6) bad luck (*zāoyù*)
composition (comp)	(18–4) complete (*jiànquán*)	(52–2) chaotic (*hùnluàn*)
valuation (val)	(43–7) valuable (*bǎoguì de*)	(24–7) vulgar (*dīliè*)

1 Martin and White (2005, 56) have finer distinctions. However, appreciative items are quite few in the data. This paper is concerned with the categories at the general level.

Narrative studies also make finer distinctions than between second-party and third-party victims (Kollareth and Russell, 2018; Hartsough et al., 2020). Narrators who finish stories are firstly distinguished from characters that play a role in stories. Secondly, narrators are further classified into heterodiegetic and homodiegetic narrators. A heterodiegetic narrator is an observer of others’ activities and a homodiegetic narrator is a character of the activity (Genette, 1980, 248). Thirdly, the interpersonal relationships between narrators and narratees and between characters are analyzed in terms of power and solidarity (Brown and Gilman, 1960).

## Research design

Hoey’s (1983) study shows that natural narrative texts include all the components of an emotional episode as proposed by the appraisal theories of emotion. Therefore, it is feasible to examine correlations between emotions and moral judgment in the context of eliciting situations from a narratological approach. The data collection took the form of structured text-completion to instruct study participants to first write down their emotional responses after reading verbal descriptions of eliciting situations and then to justify their responses.

### Materials

To be comparable with previous findings, the eliciting situations in Kollareth and Russell (2017) were used in this study with minor revisions.^2^ Kollareth and Russell (2017) employed a 3 (situations) × 3 (moral violations) design. The three situations of embezzlement, disrespect, and betrayal were used to exemplify community violations. The three situations of harm, cheating, and oppression were chosen for cases of autonomy violations. The three situations of child abuse, incest, and pathogen were selected to present divinity violations. The design provides a comprehensive coverage of the three categories of moral violations examined in the CAD hypothesis and the number of eliciting situations is suitable for the collection of open-ended data. The situations were first translated into Chinese by one of the authors and confirmed by another author through a back-translation. The two translators are both bilingual Chinese-English speakers.

### Structured questionnaire

The nine situations were first randomized and then reversed, producing two orders of presentation. For each situation, study participants were asked to have a detailed reading and imagine himself/herself in that situation witnessing the described events. A pretest (Task 1) was firstly carried out to examine whether the nine situations naturally fell into the three categories as designed by using the *Order k/n-1 with fixed K method*, which instructed study participants to: (1) use one situation as the reference point once a time; (2) choose three other situations which are most similar to the reference from the other eight; and (3) arrange the three choices according to degrees of similarity to the reference situation. Afterward, the study participants were asked (Task 2) to write down their emotional response(s) if they were in the situations and (Task 3) to justify their response(s) briefly.

### Study participants

Study participants (*N* = 59) were undergraduate students from two universities. The students are all native Chinese speakers. Thirty-one participants were female, and the remaining 28 were male. They were between 18 and 24 years old, and their mean age was 19.92. The student participants voluntarily participated anonymously, and were paid ¥20 after completing the questionnaire.

### Analysis and annotation

The analysis took the following steps. First, the similarity data from the pretest were subject to Multidimensional Scaling (MDS) to identify whether the nine situations can be classified into three categories as originally designed. Second, the responses collected from Task 2 were annotated according to affect categories (Table 1) and subject to cluster analysis after each category’s frequency was counted. Third, justifications from Task 3 were analyzed for narrators, characters, and main contents, and frequencies were counted for each item. Fourth, Chi-square tests and correspondence analyses were conducted to select items statistically associated with the situations and with the responses to the situations to explore how they correlate with each other. The codings were first made by two of the three authors independently and were agreed upon by the three authors who collaborated to identify and resolve differences and similarities.

## Results

### MDS analysis of the nine situations

Task 1 asked study participants to answer an ordered multiple-choice question. The questionnaire had two versions that differed in the order of the presentation. Version 1 was issued to 30 study participants and Version 2 to 29 study participants. The comparison of the first three choices for each situation by the two groups shows that the nine situations have the same first choice across the groups. The situations of pathogen and harm have different second choices with the second choice in one group being the third in the other group. The situations of disrespect, oppression, and embezzlement differ in their third choices. This comparison shows that the presentation order has little impact on study participants’ choices in Task 1.

**Figure 2:**
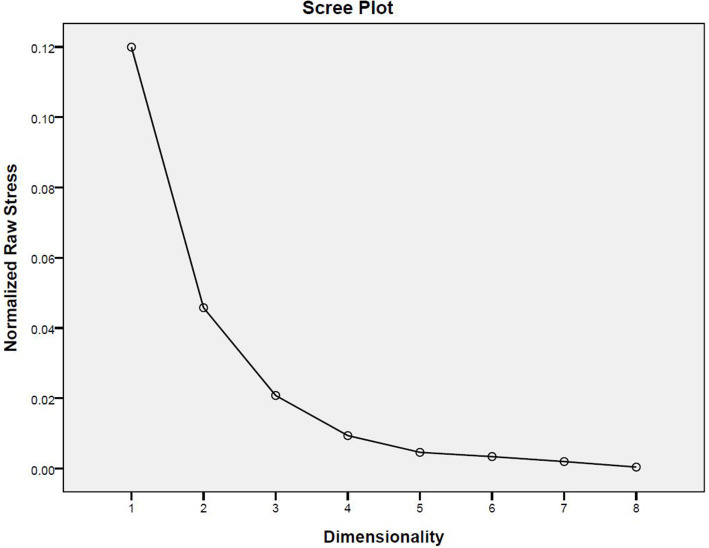
The scree plot of 1–8 dimensions.

In the MDS analysis, the Scree Plot is used to determine how many dimensions should be chosen to define the space in which stimuli is to be presented. Figure 2 shows the contributions of dimensions 1 to 8 to account for the similarity data. The first elbow appears when the second dimension is added. If 2 dimensions are chosen, the Normalized Raw Stress (0.04013) is smaller than 0.05 and D.A.F (0.95987) is larger than 0.95, which means a two-dimensional presentation can provide a good account of the similarity data.

**Figure 3:**
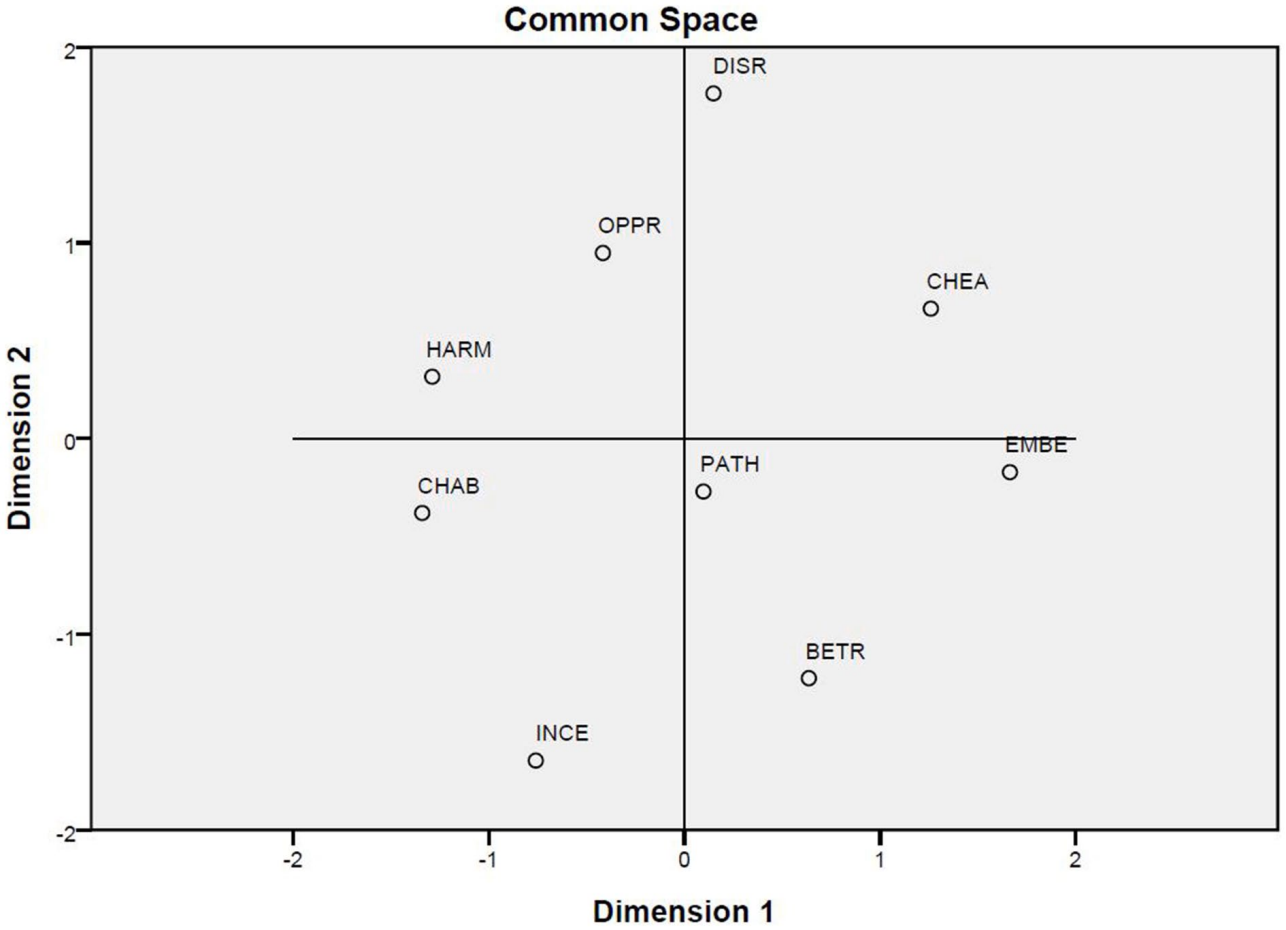
The distribution of the nine situations in a two-dimensional space.

Figure 3 visualizes the distribution of the nine situations in a two-dimensional space based on the similarity data.^3^ The scattered dots in the figure show that it is quite difficult to classify them into the three categories as originally designed.

### Emotion terms and their categories

Task 2 collected the study participants’ reactions to the nine situations. With the exception of 18 blanks, the 59 study participants contributed 513 answers, providing 686 linguistic items in token and 197 in type because some answers had two or more linguistic items. Most of the expressions realize one semantic category that are listed in Tables 1–4. However, there are correlational and combinational realizations (Song, 2015). The Chinese word *bēifèn* is a case of combinational realization, where *bēi* (sad) and *fèn* (furious) are the roots of a compound, which realize two emotions in a combinational way. Therefore, it is analyzed as the realization of two emotions. *Bǐshì* (contempt) is a case of correlational realization because it denotes a negative emotion and a negative judgment that are causally related. Therefore, it is analyzed as “-sat | o -JUDG: contempt^4^” in this study. Moreover, attitudinal items have the feature of gradability. As a result, the 197 expressions were classified into 67 categories in Appendix 1a. Their frequencies without considering gradability are summarized in Appendix 2.

**Table 5:** Sample responses and justifications and their annotations.

Examples	Emotions	Justifications
(44–1)	afraid (*hàipà*): -des; H	I am afraid that I might have a fellow like him, … (*hàipà zìjǐ yǒu zhèyàng de tóngbāo*, …) [homo; P/Cons: Social IP-IV, −gen]
	furious (*fènnù*): -sat: displeasure: anger; H	and I am furious with what he did (*duì tā suǒzuòsuǒwéi biǎoshì fènnù*) [−sat: displeasure: anger, H]. [homo; Reac: Social IP-IV, N]
(46–8)	furious (*fènnù*): -sat: displeasure: anger; H	It arouses fury to abuse one’s power to seek personal gain, and it really pisses one off that a man in power takes no care of the public. (*yǐɡōnɡmóusī běnshēn jiù zúgòu fènnù le, zàiwèizhe bùwéi rénmín kǎolǜ lǐyīng fènkǎi*) [hetero_u; Inter: Pre IP-sub CV; JUDG: t -prop-com]
	helpless (*wúnài*): -hap | s -cap: helpless; M	But we are really helpless. (*dàn women què yě zhuóshí wúnài*) [homo; Res: N, −sol]
(1–1)	alert (*jǐngtì*): -sec: disquiet: disturbed; L	To sacrifice others’ interest for his own benefits. (*tōngguò sǔnhài tārén lìyì yǐ móuqǔ gèrén lìyì*) [hetero_u; Inter: Imp IP-IV, JUDG: t -prop_auto]
	annoyed (*fǎngǎn*): -hap: antipathy: disgust; M	To sacrifice others’ interest for his own benefits. (*tōngguò sǔnhài tārén lìyì yǐ móuqǔ gèrén lìyì*) [hetero_u; Inter: Imp IP-IV, JUDG: t -prop_auto]
(30–8)	quiet (*píngdàn*): neutral: quiet; M	It is not my business, and it is far away from me. (*hé zìjǐ méi shénme guānxì, bǐjiào yáoyuǎn de shì*) [hetero_m; Comm: N, APPR: -comp]
(49–4)	sad and furious (*bēifèn*): -hap: misery; H -sat: displeasure: anger; H	Feel sad for the children, and feel furious with the violence. (*wéi értóng ér bēi, wéi bàolì ér fèn*) [hetero_u; Reac: sub CV, N; hetero_u; Reac: pre CP, N]
(17–1)	contempt (*bǐshì*): -sat | o -JUDG: contempt; M	Forget the basic principle to be human, and drag out an ignoble existence. (*wàngjì zuòrén jīběn zhǔnzé, gǒuqiětōushēng*) [hetero-u; Inter: N, JUDG: -prop_div]
(57–1)	furious (*fènnù*): -sat: displeasure: anger; H	It is betrayal to sell out one’s country fellows, and the person should be sentenced to death. (*chūmài tóngbāo yìwéi pànguó, lǐdāng sǐxíng*) [hetero_u; Inter: Social IP-CV, JUDG: -prop_com, JUDG: -law]
(6–2)	sympathetic (*tóngqíng*): -hap | o -norm: sympathy; M	Incest is rather morally unacceptable, and it probably results from psychological diseases. (*luànlún bǐjiào bù wéi shèhuì lúnlǐ jiēnà, kěnéng shì zìshēn huàn yǒu xīnlǐ lèi jíbìng*) [hetero_u; Inter: Imp IP, JUDG: -prop_div & hetero_u; Comm: Imp IP, +acc]
(43–7)	intolerable (*wúfǎ rěnshòu*): -sat: displeasure: anger; L	Freedom is the most valuable thing. (*zìyóu shì zuì bǎoguì de*) [hetero_u; Comm: N, APPR: +val]
(47–8)	furious (*fènnù*): -sat: displeasure: anger; H	It threatens people’s interests, and leads to injustice. (*sǔnhài gōngmín lìyì, zàochéng shèhuì bùgōng*) [hetero_u; Cons: CV, −auto]
(1–6)	alert (*jǐngtì*): -sec: disquiet: disturbed; L	The social value system gradually collapses. (*shèhuì jiàzhíguān zhújiàn gēliè*) [hetero_u; P/Cons: N, −com]
(37–2)	vomitous (*fǎnwèi*): -hap: antipathy: disgust; M	Junior–senior hierarchy and biological knowledge make it clear to me that this type of act will lead to harmful consequences. (*zhǎngyòuyǒuxù jí xuéxí de shēngwù zhīshi shǐ wǒ zhīdào zhèyàng huì chǎnshēng de èxìng hòuguǒ*) [hetero_u; Cons: N, −div]
(35–8)	flat (*lěngmò*): -sat: ennui; H	The phenomenon is prevalent. The person should be sent to court. That’s it. (*shuò jiàn bù xiān, shòu fǎlǜ zhìcái jíkě*) [hetero_u; Inter: N, JUDG: +norm & hetero_u; Res: N, +sol]

The general findings show the study participants’ responses to the nine situations greatly vary. In general, the responses consist of both emotional and non-emotional responses. The non-emotional responses are of two subcategories: understanding (“under” for short) and neutral. The responses in the neutral category are not emotional and are not classifiable according to gradability. The emotional responses are of three categories in terms of value, that is, positive, negative, and mixed. Positive responses are primarily in the category “+sat: interest,” and the mixed emotional response has one case in the category, that is, *qíngxù fùzá* (mixed emotions). Negative emotional responses have the largest proportion of responses. The top three responses include anger (24.8%), disgust (20.7%), and contempt (in the category of “-sat | o -JUDG: contempt”; 7.7%), totaling 53.2% of all responses. The figures in Appendix 1b visually present the responses to each situation, which show that some situations, for example those of embezzlement and betrayal, mainly trigger one emotional response, while others, like those of disrespect and cheating, can lead to several responses with almost equal frequencies.

**Figure 4:**
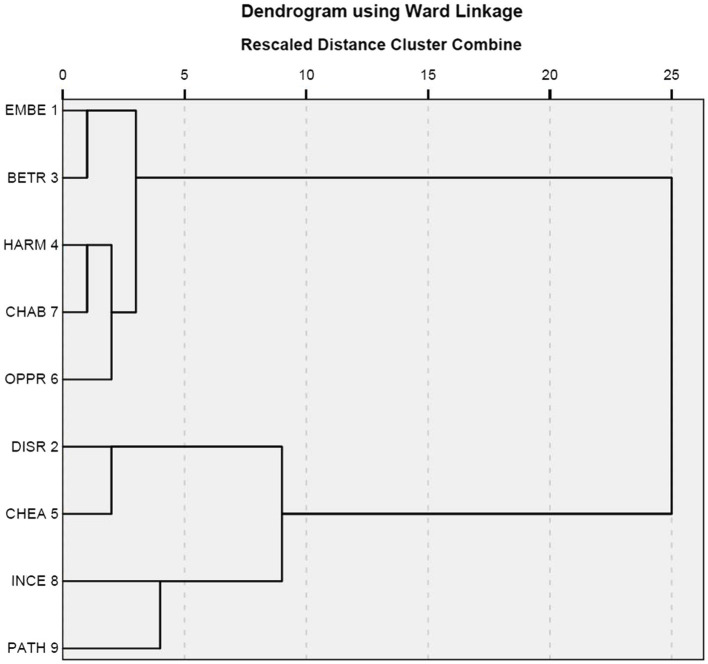
Cluster analysis on all responses.

**Figure 5:**
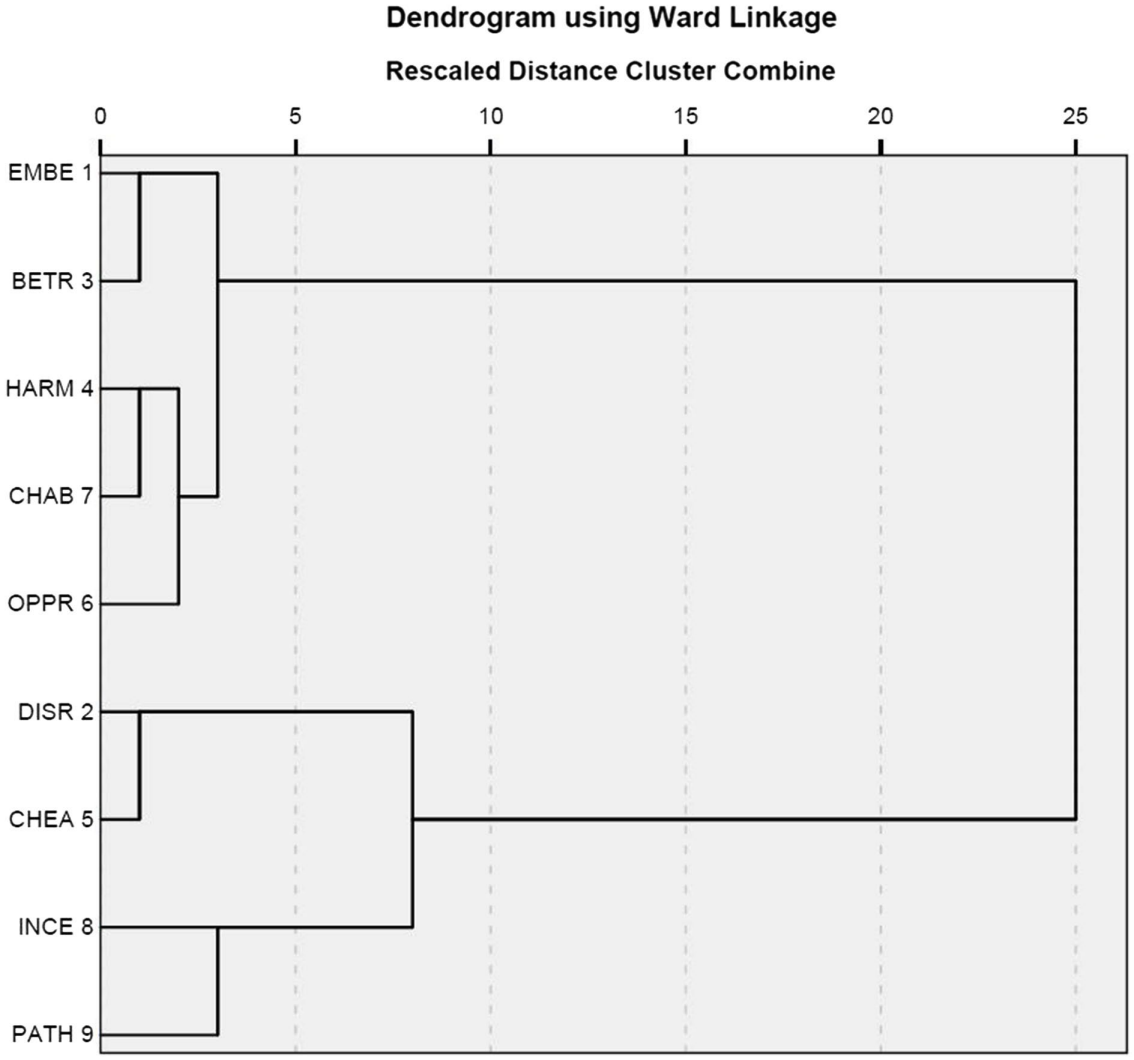
Cluster analysis on emotional responses.

Figures 4, 5 present the results of hierarchical classification of the nine situations firstly by using as data all the responses (Figure 4) and secondly by using the emotional responses (Figure 5). These two results were somewhat similar with a slight difference concerning the closeness between the situations of incest and pathogen. In addition, the classifications of the situations into two broad categories and four finer categories are similar to the result reported in Figure 3.

### Narrators, characters, and bases in justifications

Task 3 required study participants’ justification for their response(s). An examination found that one response was not justified and the justifications by study participant number 14 were invalid as she copied herself. The number of valid justification texts was 503, and the final number of justifications was 829 as some justification texts contain two or more justifications. As a part of narrative, justifications could be analyzed in terms of narrators, characters, and main content (which, for the convenience of discussion, will be referred to as bases with reference to Hoey’s (1983) theory). An analysis of the justifications with reference to the narratological concepts introduced in section *The narratological approach and its concepts to be used* show that a full description of a justification can be made by employing the system network in Figure 1. Table 5 provides some sample descriptions. The frequencies of each category of narrator, character, and basis are given in Appendices 3a-c.

The first system is referred to as “strategy” with two features: opt in and opt out. It works together with the other systems to account for the presence (i.e., opt in) or absence (i.e., opt out) of a system feature or a system.

The system of narrator distinguishes heteroglossic from homoglossic narrators, recapitulating the distinction between second-party and third-party victims in previous studies (for example, Kollareth and Russell, 2018; Hartsough et al., 2020). Appendix 3a shows that, instructed to imagine themselves in the given situations, 93.6% of the study participants reacted to the situations as observers (i.e., heteroglossic narrators), while merely 6.4% gave their responses as characters in the described event (that is, homoglossic narrators; Examples (44–1) and (46–8) in Table 5). Heteroglossic narrators can be divided into unmarked (hetero_u in short; Example (1–1) in Table 5) and marked (short as hetero_m) categories. The marked category employs a disinvolvement strategy, distancing themselves from the event [Example (30–8) in Table 5], and it has only eight cases. Narrators are always present in justifications, which means the opt-in choice in the system of strategy is always motivated.

The system of character, working together with the system of strategy, accounts for the distinction between act-orientation and character-orientation (Giner-Sorolla and Chapman, 2017) and provides richer tools to analyze characters. If the opt-out strategy is taken, it means the justification focuses on the act and its characters will be annotated as N (i.e., none). Appendix 3b shows that 41.9% of justifications were act-orientated. The combination with the opt-in feature of the strategy system implies the justification mentions at least one character. Characters were analyzed in terms of types, roles, and interpersonal relationships. The type subsystem consists of two features, namely individual (I) and community (C), to reflect the distinction between autonomy and community in the CAD hypothesis (Rozin et al., 1999). The role subsystem explains the traditional distinction between protagonists and antagonists. However, the paper chooses to employ perpetrators (P) and victims (V) because the nine situations are all about violations of moral codes. The other characters in the events are annotated as “other.” The interpersonal relationship follows Brown and Gilman’s (1960) theory and consists of two dimensions, namely power and solidarity. Both dimensions are assigned three values as indicated in Figure 1. With reference to the three subsystems, the characters in Examples (44–1), (46–8), and (1–1) in Table 5 can be analyzed as Social IP-IV, Pre IP-sub CV, and Imp IP-IV. Appendix 3b shows that the characters are greatly diversified and impersonal individual perpetrators (imp IP; 11.5%) and predominant individual perpetrators (pre IP; 7.1%) are the most frequent choices.

The basis system is more complicated than the other three systems and it has four choices at the first level. This system helps us to analyze the main justification content. Reaction refers to justification cases in which a study participant takes an emotional reaction as something spontaneous and just writes down his/her reaction(s). Example (49–4) in Table 5 is such a case in which the first emotion is justified only by mentioning the victim. The eliciting situation which is followed only by some reaction(s) is referred to as remarkable event in Martin and Rose (2008, 52). Appendix 3c shows that 7% (0.24% + 6.76%) of justifications are in this category.

Interpretation refers to justification cases where a study participant bases his/her emotional responses on either explicit or implicit judgment. Martin and White (2005) divide judgment into the categories of social esteem and social sanction (Tables 2 and 3). Our data showed that jurisdiction and neutral judgment should be added, and their subcategory of propriety should be further divided into autonomous (auto), communal (com), and divine (div) according to Shweder et al.’s (1997) triple classification of ethics. The eliciting situation which is followed by an interpretation is referred as incident in Martin and Rose (2008, 52). Among all the justifications, 73.34% are in this category. According to Appendix 3c, the three subcategories of propriety, corresponding to the three types of moral violations in Rozin et al. (1999), contributed about 43% of all bases (Table 5; 1–1), (17–1), and (57–1).

Comment refers to justification cases where a study participant makes his/her justification by making some comments. It can be further divided into accountability to explain whether the event is acceptable [Example (6–2) in Table 5] and appreciation to show how the event is appreciated as a thing [Example (43–7) in Table 5]. The eliciting situation which is followed by a comment is referred to as event description in Martin and Rose (2008, 52). Accountability contributes to approximately 11% of all bases.

The last choice is to interpret the eliciting situations as complicating actions (Martin and Rose, 2008, 52) and provide their subsequent consequences or possible resolutions, trying to build the eliciting situations into stories with a conclusion. Consequences are classifiable according to the three types of ethics mentioned above (see Example (47–8) for rights violation, Example (1–6) for community preservation, and Example (37–2) for the divinity code, in Table 5), and consequences for the autonomy code make the largest contribution (14.35%). Regarding resolution, Example (35–8), in Table 5 offers an illustration, suggesting that the person be taken to court.

### Correspondence analysis of situations, emotions, and their justifications

The presentation order, as previously discussed, had little impact on the study participants’ choices in Task 1, and the same applies to Tasks 2 and 3 as the Chi-square tests of the two groups by treating narrators, characters, and bases as nominal variables show that no *p* values were smaller than 0.05.

The relationships among different elements are examined by using the method of correspondence analysis. This method has two requirements. The more basic one is that 80% of the cells in the table formed by row and column variables have an expected count no smaller than 5. The second is that there is a relationship between the variables considered. To satisfy the first requirement, the examinations of responses and of the bases have to be limited to the top three emotional responses and the three subcategories of propriety (see Appendix 3c’ where subcategories of basis are further combined), and the limitations fortunately still make it possible to examine the CAD hypothesis. In addition, the subcategories of characters have to be combined as in Appendix 3b’. Finally, the narrator variable has to be ignored because two of the three subcategories are extremely limited in number.

Table 6 summarizes the results of the bi-dimensional Chi-square tests of these variables and their Craver’s Vs. The *p*-values, all smaller than 0.05, indicate that there is a relationship between each pair of variables. Cramer’s *Vs* show that the degrees of association between each pair vary from one another. That between the variables of emotion and participant is between small and moderate (0.1–0.3), while the others are moderate (0.3–0.5).

**Table 6:** Chi-square test results and Cramer’s V of variable pairs.

Variables	Chi-square test results			Cramer’s V
	χ^2^	df	*p*	
Situation vs. emotional response	102.661	16	0.000	0.412
Basis vs. situation	129.574	16	0.000	0.462
Basis vs. emotional response	80.725	4	0.000	0.365
Emotional response vs. character	28.135	10	0.002	0.215
Basis vs. character	77.829	10	0.000	0.358
Character vs. situation	286.755	40	0.000	0.435

**Figure 6:**
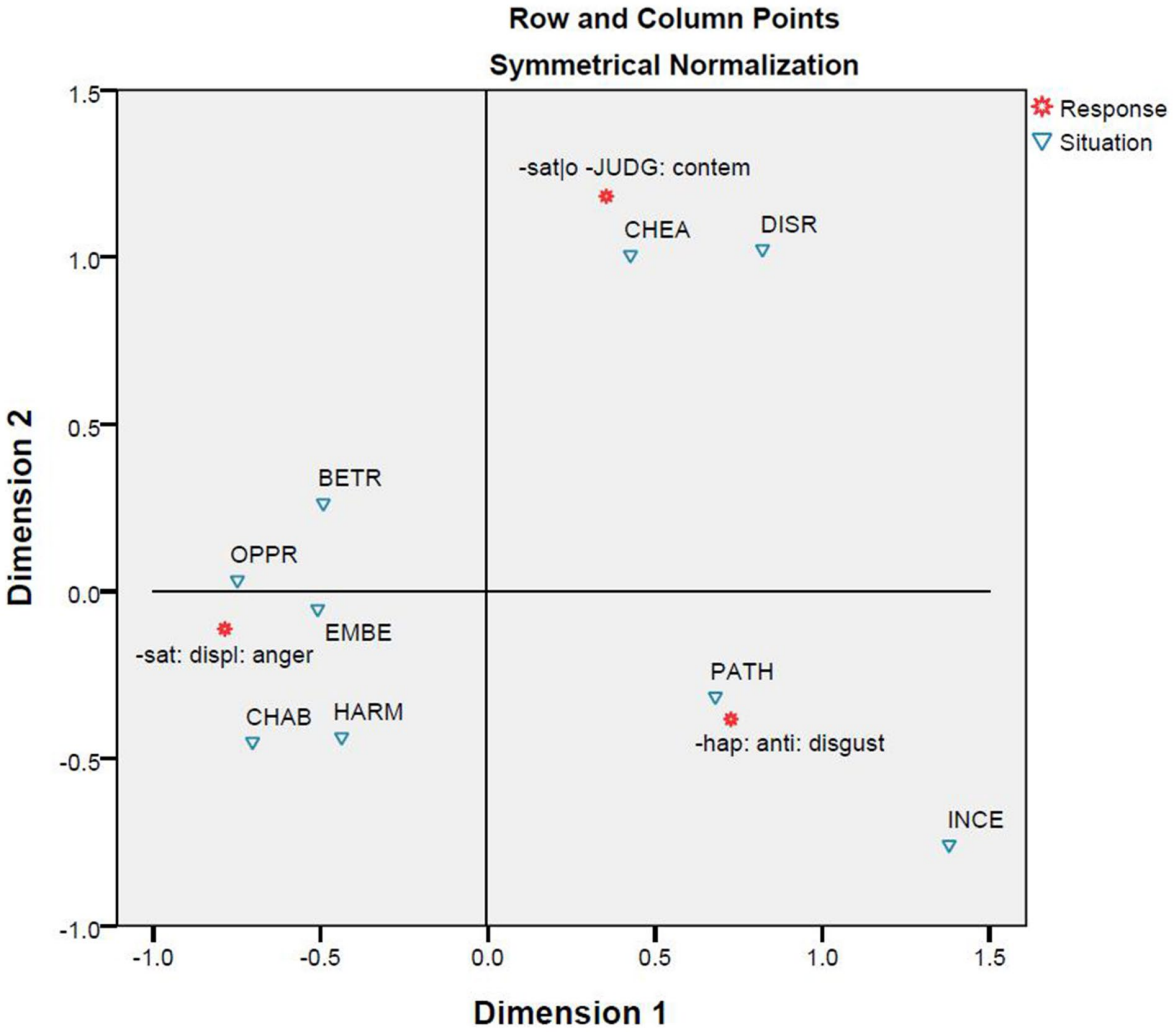
Correspondence analysis between situation and response.

The correspondence analysis between the nine situations and the three emotions (Figure 6) is similar to the cluster analyses whose results are reported in Figures 4, 5 in that all aim to classify situations based on responses. However, this correspondence analysis differs from the two cluster analyses in basing its result on a smaller part of the responses. A comparison of Figures 4, 5 shows that the narrowing down from all responses to emotional responses has little influence on the results. Nevertheless, a comparison of Figure 6 with Figures 4, 5 shows that further narrowing down to the three emotions leads to some differences. However, the differences are minor. Figures 4–6 all suggest two broad categories can be identified. One broad category is formed out of the subcategory consisting of the situations of cheating and disrespect and the subcategory containing the situations of pathogen and incest. The other broad category is formed from the other five situations. In Figures 4–6, the distances between the situations of child abuse and harm and between the situations of betrayal and embezzlement are similarly close; however, the situation of oppression is closer to those of child abuse and harm in Figures 4, 5 but becomes closer to those of embezzlement and betrayal in Figure 6. Moreover, Figure 6 explicitly shows that the situations of pathogen and incest are more likely to trigger the emotion of disgust, while the situations of cheating and disrespect tend to evoke the emotion of contempt. The remaining five situations could possibly provoke the emotion of anger.

**Figure 7:**
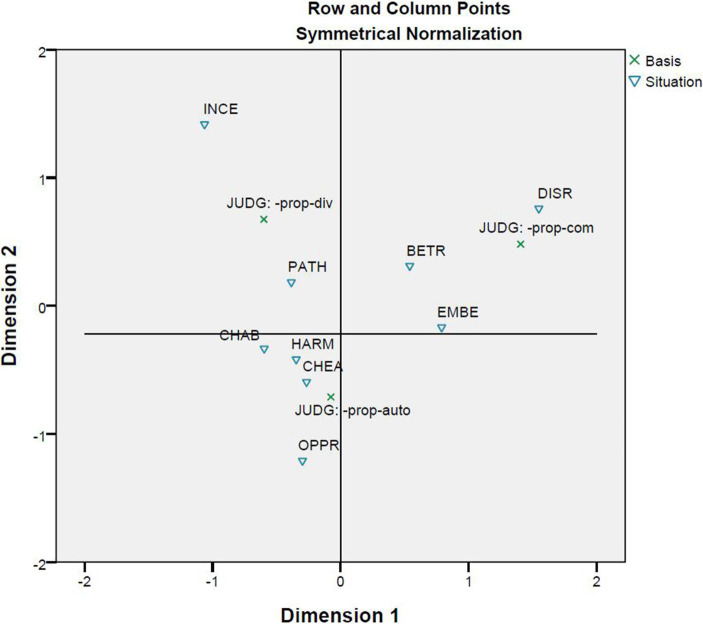
Correspondence analysis between basis and situation.

The correspondence analysis between basis and situation (Figure 7) shows that the situations of disrespect, betrayal, and embezzlement are more likely to be interpreted as cases of community violations, while the situations of incest and pathogen are cases of divinity violations. The situation of child abuse should be close to those of incest and pathogen as designed by Kollareth and Russell (2017). However, it is closer to the situations of harm, cheating, and oppression, and these four situations instantiate autonomy violations.

**Figure 8:**
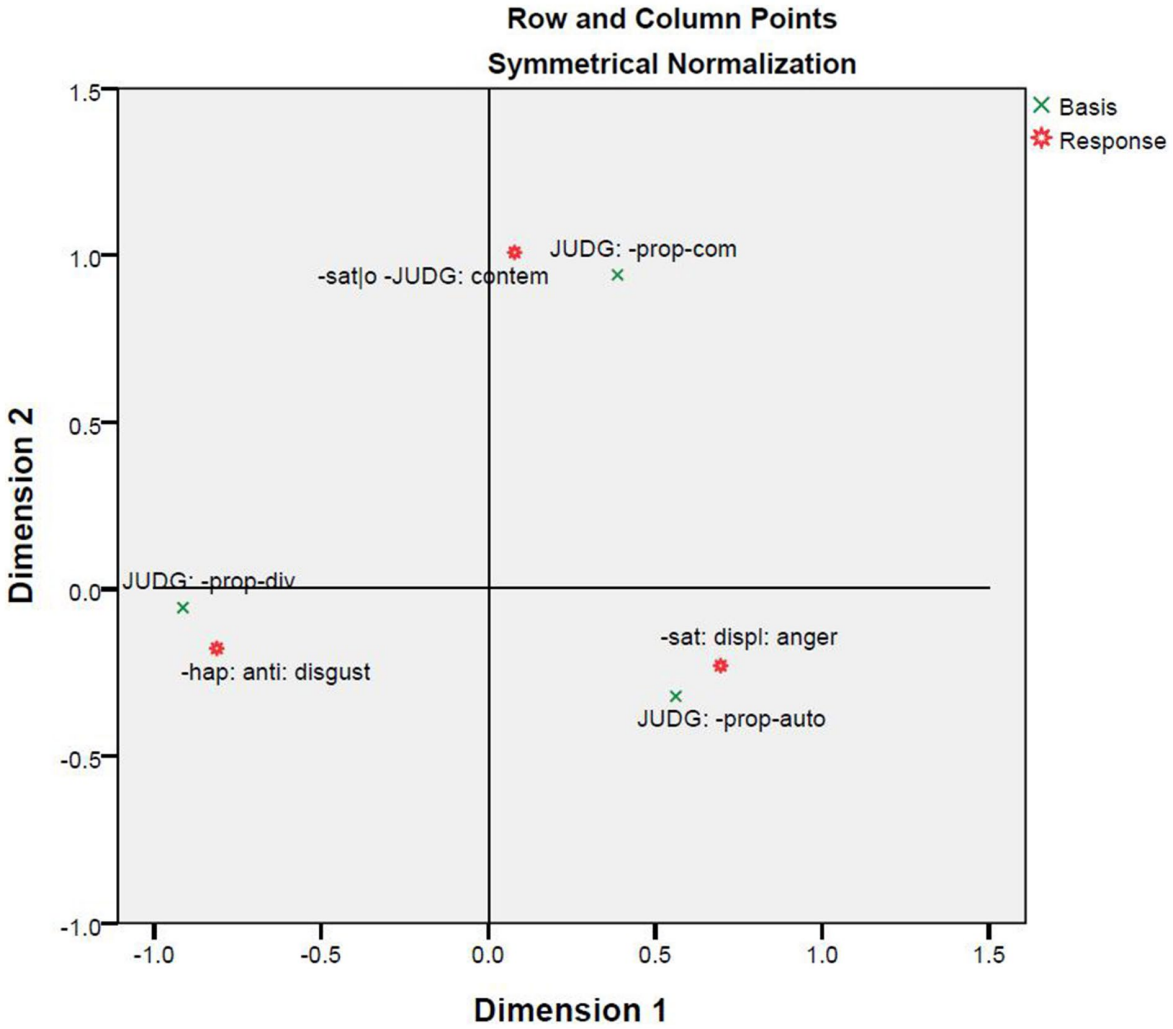
Correspondence analysis between basis and response.

**Figure 9:**
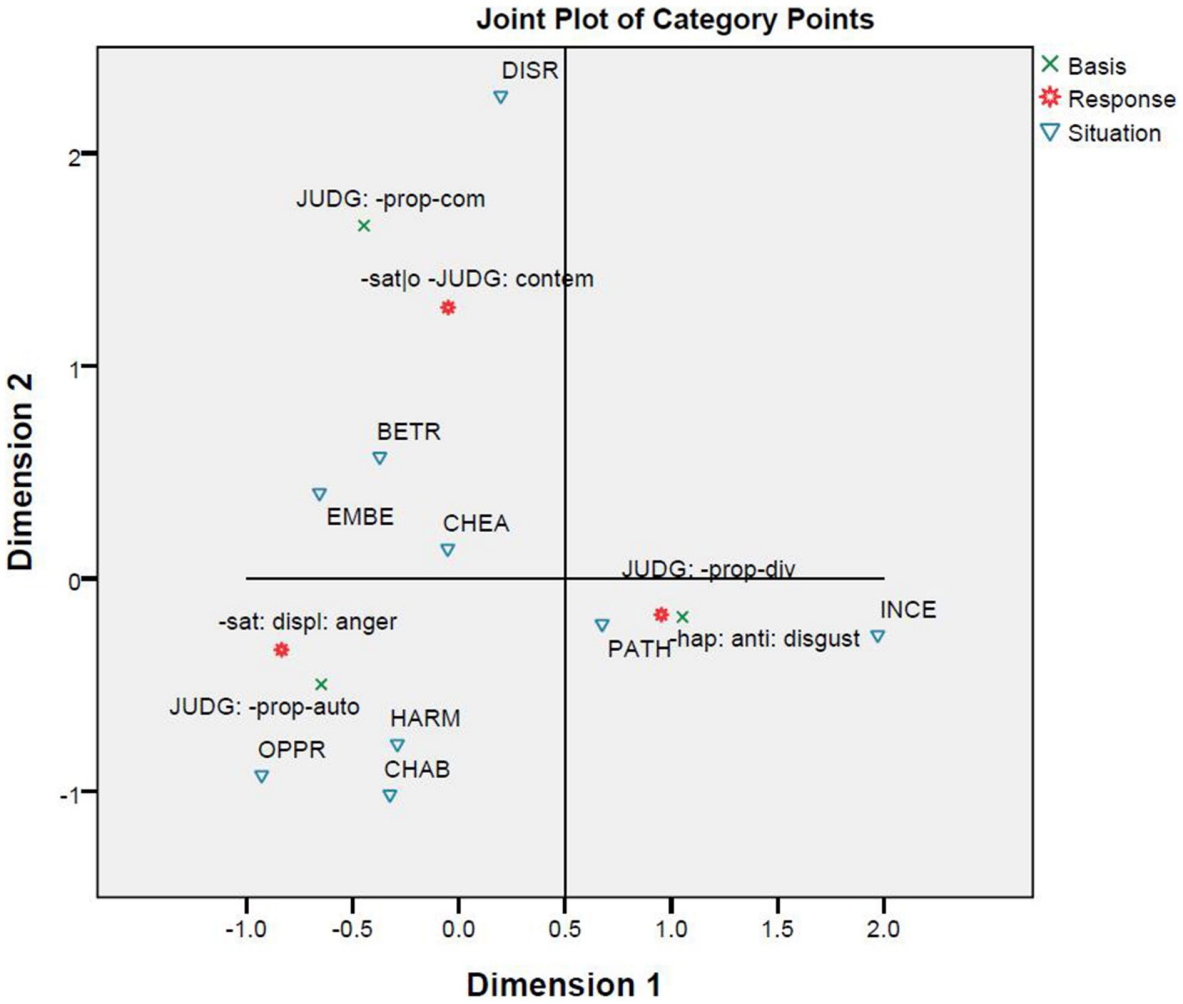
Correspondence analysis among basis, response and situation.

The correspondence analysis between basis and response provides support to the CAD hypothesis because the violations of ethics and their predicted emotions are closely correlated (Figure 8). Figure 9 brings basis, response, and situation into one correspondence analysis. The figure shows there are three clusters: The first is formed out of the emotion of disgust, divinity violations, and the situations of incest and pathogen; the second contains the emotion of contempt, community violations, and the situations of betrayal, embezzlement, and disrespect; and the third results from the correlation among the emotion of anger, autonomy violations, and the situations of oppression, harm, and child abuse. A comparison of Figures 7–9 shows that the correlations in the three clusters are quite stable. The only exception is that the situation of cheating correlates with autonomy violations in Figure 7 but with community violations in Figure 9.

**Figure 10:**
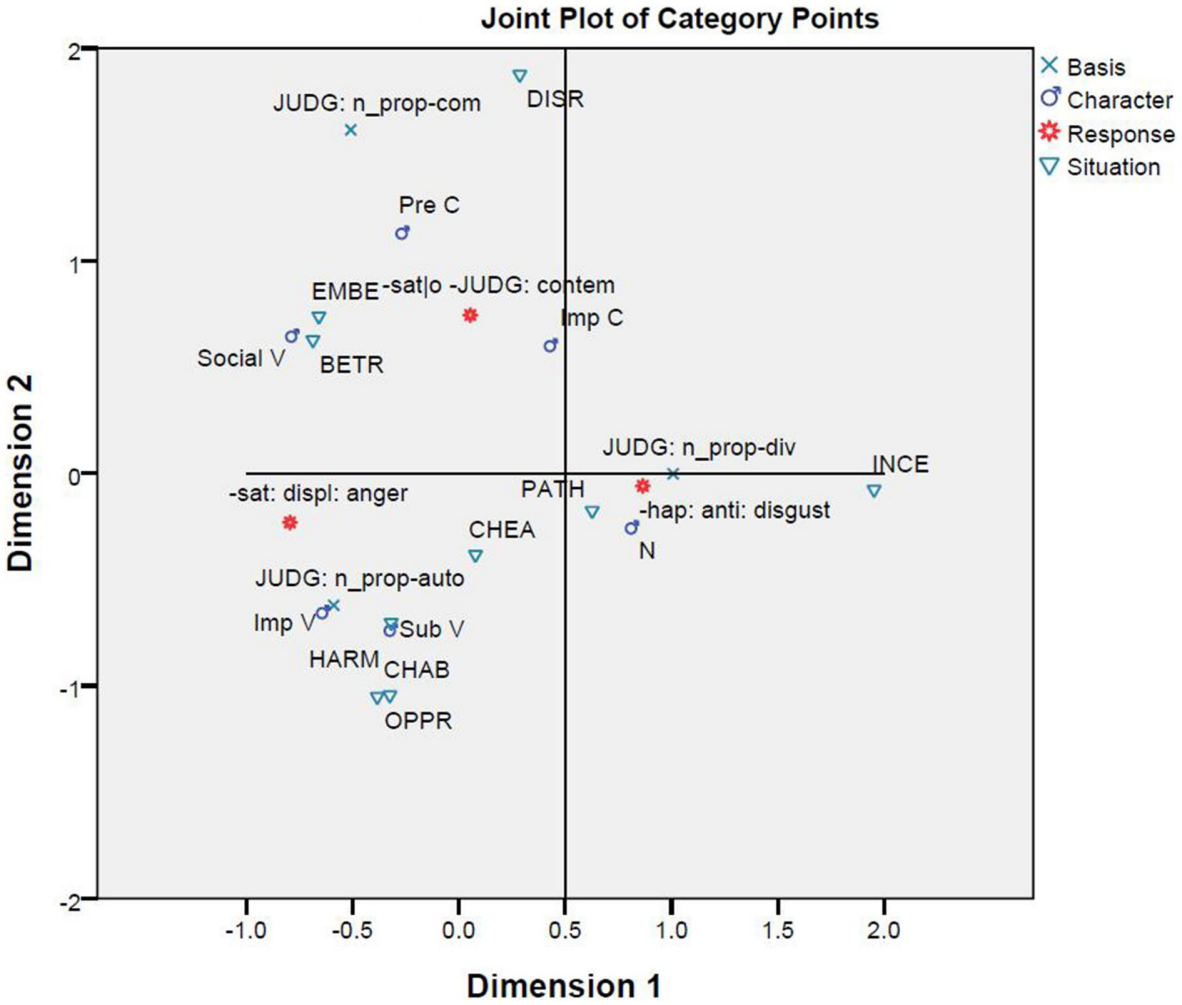
Correspondence analysis among basis, character, response, and situation.

Additionally, the last correspondence analysis brings the variable of character into consideration (Figure 10). Three clusters can be identified, although the results must be taken cautiously because the Chi-square test between situation and character might have some misleading results due to the condition that more than 20% of the cells have expected counts less than 5. The cluster easiest to identify is formed out of the situations of incest and pathogen, the emotion of disgust, divinity violations, and the combination of the opt-out feature in the strategy system and the character system. Example (37–2) in Table 5 is a good illustration, where the vomitous reaction (a case of disgust) to the situation of incest is justified by a basis featuring a divinity violation (JUDG: -prop_div) without mentioning any specific character (i.e., N). Example (10–3) in Table 7 is a case where the negative judgment in terms of divinity justifies an upgraded disgust in the situation of pathogen.

**Table 7:** Cluster examples.

Example number	Situation	Emotion	Justification
(10–3)	pathogen	abominable (*yànwù*) [−hap: antipathy: disgust, H]	The behavior that harms other people’s health is among the most vulgar. (*bù gùjì tārén jiànkāng ānquán de xíngwéi zuìwéi dīxià*) [hetero_u; Inter: N, JUDG: -prop_div]
(30–9)	harm	furious (*fènnù*) [−sat: displeasure: anger, H]	In no situation should one harm another person. (*wúlùn fāshēng shénme shì, shānghài tārén shì bù yīnggāi de*) [hetero_u; Inter: Imp_V, JUDG: -prop_auto]
(19–9)	harm	furious (*fènnù*) [−sat: displeasure: anger, H]	Feel absolutely furious with physical violence, especially the one done by the stronger upon the weaker. (*duì rénshēn gōngjī, tèbié shì shìqiánglíngruò de xíngwéi fēicháng fènnù*) [hetero_u; Inter: Sub_V, JUDG: -prop_auto]
(57–7)	oppression	furious (*fènnù*) [−sat: displeasure: anger, H]	Everyone is born equal, and no one has the right to interfere in others’ issues. (*rén sheng ér píngděng, tārén wúquán gānshè biérén zìyóu*) [hetero_u; Inter: Imp_V, JUDG: -prop_auto]
(38–7)	oppression	furious (*fènnù*) [−sat: displeasure: anger, H]	To wall one’s mind aims to train an obedient follower by distancing him/her from the community. (*jìngù sīxiǎng, biànxiàng de yòng zìyóu móulì, bù róngrù qúntǐ*) [hetero_u; Inter: Sub_V, JUDG: -prop_auto]
(40–4)	Child abuse	furious (*fènnù*) [−sat: displeasure: anger, H]	To maim others in order to satisfy oneself, which is unforgivable. (*wèile zìjǐ de lìhài duì tārén jinxing cánhài, bùkě yuánliàng*) [hetero_u; Inter: Imp_V, JUDG: -prop_auto]
(48–4)	Child abuse	furious (*fènnù*) [−sat: displeasure: anger, H]	It is to harm children’s health and even threaten their life. Furthermore, children are so vulnerable. (*qīnfàn háizi shēngmìng jiànkāng quán, érqiě duì háizi zhème ruòxiǎo de gètǐ*) [hetero_u; Inter: Sub_V, JUDG: -prop_auto]
(20–5)	cheating	furious (*fènnù*) [−sat: displeasure: anger, H]	To sacrifice others’ interest for one’s own satisfaction. (*sǔnhài tārén lìyì yǐ mǎnzú zìjǐ*) [hetero_u; Inter: Imp_V, JUDG: -prop_auto]
(10–1)	betrayal	look down on (*mièshì*) [−sat|o -JUDG: contempt]	It is selfish and ruthless to betray one’s country fellows. (*chūmài tóngbāo shì zìsī zìlì wúqíng wúyì de tǐxiàn*) [hetero_u; Inter: Social_V, JUDG: -prop_com]
(19–8)	embezzlement	despise (*bǐshì*) [−sat|o -JUDG: contempt; M]	Government expenses are from the taxes contributed by every citizen, but officials put them into their pockets. I feel that my rights are infringed. (*gōngkuǎn shì yóu měigè gōngmín gòngxiàn de, guānyuán jùwéijǐyǒu. Gǎndào zìjǐ quánlì shòudào qīnfàn*) [homo; Inter: Pre_P, JUDG: -prop_com]
(20–6)	disrespect	disdain (*bǐyí*) [−sat|o -JUDG: contempt; M]	A person should not be forced to give up his/her seat to others. The refusal to do so only makes one feel morally uneasy. (*bùnéng qiángzhì yāoqiú tārén ràngzuò, zhǐshì zài dàodégǎn shang bù shūfu*) [homo; Inter: Imp_P, JUDG: -prop_com]

Another cluster is composed of the situations of harm, oppression, and child abuse, the emotion of anger, autonomy violations, and impersonal and subordinate victims. Examples (30–9), (10–9), (57–7), (38–7), (40–4), and (48–4) in Table 7 share the response of fury (*fènnù*) [−sat: displeasure: anger, H] and the basis of negative judgment in terms of autonomy violations (JUDG: -prop_auto), and show the combinations of the three situations and the two categories of characters. To this cluster, the situation of cheating could be added, which, however, differs from the other three in that its participants in the justification text are mostly in the subcategory of impersonal victims [see Example (20–5) in Table 7].

The third cluster has its core formed by the situations of betrayal and embezzlement, the emotion of contempt, community violations, and characters such as social victims (Social_V), predominant perpetrator (Pre_P), and impersonal perpetrator (Imp_P). Example (10–1) in Table 7 illustrates how contempt is justified in community ethics in the situation of betrayal that features a social victim, and Example (19–8) in the same table shows how contempt is similarly justified in the situation of embezzlement that focuses on a predominant perpetrator. The situation of disrespect could be loosely included in this cluster, although it is quite distant from the other two situations. Example (20–6) in Table 7 is a case where the situation of disrespect, the emotion of contempt, the basis of community violations, and an impersonal perpetrator form a constellation.

## Discussion and conclusion

This study’s results are mainly based on descriptive statistics of the open-ended data collected through a structured questionnaire and on correspondence analyses. The descriptive statistics show that the study participants’ responses and their justifications are both diverse, including non-emotional responses [for example, quiet (*píngjìng*)] and non-appraisal bases (for example, accountability). The responses and the bases of justifications can be classified into 25 and 28 categories, respectively, (Appendices 2, 3c). Among them, the categories of anger (24.8%), disgust (20.7%), and contempt (7.7%) add up to more than a half of responses, and negative judgments based on the three moral codes of autonomy (30.03%), divinity (18.1%), and community (11.82%) occupy about 60% of the bases. Moreover, the percentages of the three emotions and the three moral violations match quite well. Specifically, the lowest percentage of contempt and its big differences with those of anger and disgust suggest contempt, rather than disgust, is not a typical social emotion (*cf.* Piazza and Landy, 2020). This atypicality is accountable because contempt differs from the other two emotions in that it is a case of negative satisfaction casually related to negative judgment of others. Furthermore, the results reported in Figures 3–5 show that the nine situations are not perfect instantiations of the three moral violations. The results summarized in Appendix 1a and visualized in Appendix 1b show that the fixed set of candidate emotions in Rozin et al. (1999) is inadequate.

The correspondence analyses were conducted step by step. A comparison of the results leads to more interesting points in addition to the ones they make clear when standing alone. The first correspondence analysis focuses on the relationship between the nine situations and the three moral emotions (Figure 6). Using the same set of situations with minor differences, the analysis and Kollareth and Russell (2017) have both similarities and differences. The two studies similarly observe the correlations between the emotion of disgust and two cases of divinity violations, namely the situations of pathogen and incest, and between the emotion of anger and two cases of autonomy violations (i.e., the situations of harm and oppression), two cases of community violations (i.e., the situations of embezzlement and betrayal), and one case of divinity violations (that is, the situation of child abuse). The present study differs from the one by Kollareth and Russell (2017) in finding that the situation of cheating (a case of autonomy violations) and the situation of disrespect (a case of community violations) both elicit the emotion of contempt rather than the emotion of anger. However, the statistics in Appendix 1b show that anger and contempt are in fact almost equally recorded within the context of cheating, and that anger is among the first four emotional reactions to the situation of disrespect. It means that the categorical differences reported in these two studies might not be so substantial, and are tolerable if their design differences are further considered. Kollareth and Russell (2017) recruited study participants from three cultural groups and collected data by using the method of forced choices from a fixed set, but the present study recruited study participants from a fourth cultural group and collected open-ended responses as data.

The second and third correspondence analyses are of the relationships between basis and situation (Figure 7) and between basis and response (Figure 8). Kollareth and Russell (2017) challenged the CAD hypothesis based on correlations between situations and responses that were previously discussed. However, the results reported in Figure 7, together with those in Figure 3, indicate that the nine situations do not fall into the categories as designed. Moreover, Figures 3–5 show that there is a correlation between study participants’ perception of the situations and their reactions to the situations. Furthermore, Figure 8 shows a moderate correlation between the three emotions and the three types of bases that are defined by the three types of moral violations. Therefore, Kollareth and Russell’s (2017) challenge seems to be supported if only the relationships between situations and emotions are considered. However, this does not hold when the categorization of the situation is taken into account because the classification of the nine situations into three categories is not reported in the present study.

The fourth correspondence analysis brought together situations, responses, and bases, and the fifth correspondence analysis added characters. A comparison of Figures 6–10 shows that most clusters are quite persistent, while two of them are prone to changes. The persistent clusters include the correlations between the emotion of disgust and the situations of pathogen and incest, and between the emotion of anger and the situations of harm, oppression, and child abuse; both emerge in Figure 6 and persist when the bases and characters are added into the analysis (Figures 9, 10). Another persistent cluster is between the three emotions and the three types of moral violations as shown in Figures 8–10. The two changeable clusters include the correlations between the emotion of anger and the situations of embezzlement and betrayal and between the emotion of contempt and the situation of cheating, both established in Figure 6. Between the changeable clusters, the first changes when bases are taken into consideration (Figure 9) and becomes fixed when the character variable is later introduced into the analysis. The second collapses only when characters are finally introduced (Figure 10). Moreover, the relation between the emotion of contempt and the situation of disrespect is in-between because the close correlation in Figure 6 becomes quite looser in Figures 9, 10.

The findings lead to two conclusions. Firstly, between moral judgment or evaluations of situations (the bases of justification in this study) and situations, the former provides a more reliable account of the elicitation of moral emotions. This finding can account for both persistent and changeable relationships between situations and emotions. The persistent cases probably arise from the fact that some situations are prone to a specific appraisal, while the changeable cases are due to the fact that other situations are subject to diverse judgment or evaluations. Therefore, the key tenet of appraisal theories of emotion might be too strong in holding that the same event may be diversely evaluated because events differ in their liability to different evaluations. Moreover, some events might trigger spontaneous reactions without any evaluation (see Appendix 3c). Therefore, the selection of eliciting situations should take possible and different evaluations into consideration. Secondly, moral emotions are conditioned by multiple factors. Hence, the failure to bring into account the variables of basis and character results in biased understandings of moral emotions. This can explain the following conflict between the findings of the present study and the findings in Giner-Sorolla and Chapman (2017). Giner-Sorolla and Chapman (2017) argued that disgust was largely triggered by a bad moral character. However, Figure 10 shows that disgust is an emotional reaction to the situations of incest and pathogen under the condition that they are morally judged in terms of divinity violations and there are no characters involved.

The last issue to be addressed is the fallacy of false dilemma that likely happens when the method of forced choices is employed. The diverse responses (Appendix 2) suggest it might be possible in previous studies. However, the influence of the fallacy is limited if it does exist, since the three social emotions occupy over a half of the responses. Although the other responses are rich in categories, they are quite small in percentage. More importantly, no significant differences are found in the comparison of Figures 4–6 that record the results based on data narrowing down from all responses to emotional responses and finally to the three emotions.

This study has two theoretical contributions. Firstly, it supports the CAD hypothesis if moral judgment (interchangeably evaluations in the paper) is treated as the essence of moral violations because there is a moderate correlation between the three bases defined in terms of the three moral codes and the three moral emotions, which is conditioned by the variable of character in the situations. Secondly, the study elaborates about the appraisal theories of emotion by providing a framework (Figure 1) to analyze the appraisal component between eliciting situations and emotions and indicating that it is too strong to claim that an event is prone to different evaluations. Nevertheless, the first contribution suggests that further studies are required to better understand the three social emotions in specific and emotion in general. Characters are one of the variables that have an influence on moral emotions. Some factors that have been mentioned in previous studies such as intentionality (Kollareth and Russell, 2018) and the distinction between second-party and third-party norm violations (Hartsough et al., 2020) were not explored in the present study due to a lack of data. Furthermore, other responses with the exception of anger, contempt, and disgust were not discussed also due to a lack of data. The present study is exploratory. Future studies could have a larger data to further investigate other variables and other emotions.

## Data availability statement

The original contributions presented in the study are included in the article/Supplementary material, further inquiries can be directed to the corresponding author.

## Ethics statement

Ethical review and approval was not required for the study on human participants in accordance with the local legislation and institutional requirements. Written informed consent for participation was not required for this study in accordance with the national legislation and the institutional requirements.

## Author contributions

CS designed the research and wrote the paper. XR helped with the design of the questionnaire, the data collection, and annotation. NX helped with the translation of verbal transcriptions of eliciting situations, the data collection, and annotation. All authors contributed to the article and approved the submitted version.

## Funding

The research is sponsored by a grant for young researchers from the Humanities and Social Sciences Youth Foundation of the Ministry of Education, China (grant no. 16YJC740061).

## Conflict of interest

The authors declare that the research was conducted in the absence of any commercial or financial relationships that could be construed as a potential conflict of interest.

## Publisher’s note

All claims expressed in this article are solely those of the authors and do not necessarily represent those of their affiliated organizations, or those of the publisher, the editors and the reviewers. Any product that may be evaluated in this article, or claim that may be made by its manufacturer, is not guaranteed or endorsed by the publisher.

## Supplementary material

The Supplementary material for this article can be found online at: https://www.frontiersin.org/articles/10.3389/fpsyg.2022.1019485/full#supplementary-material

## Footnotes

^1^The annotation of responses and justifications was done by using the abbreviations introduced in brackets in Tables 1–4 and Figure 1. In this case, "-" means "negative," and "sat" is short for "satisfaction" (Table 1). The codes not introduced in Tables 1–4 and Figure 1 are explained in footnotes. Here "JUDG" is short for "Judgement," and "o" means others, in contrast with "s" (self). "O -JUDG" means the negative judgment about others.

^2^First, in the "Embezzlement" situation, the original "governor, whom you had voted for" was changed into "a government official of your city" because China’s political system is based on the system of the People’s Congress. The second change was in the "Oppression" situation where "US/India" was replaced by "China." The third was in the "Incest" situation where "his elder sibling" was changed into "one of his family members," because most study participants were the only child in their families due to China’s previous "one-child" policy.

^3^To facilitate the presentation, the situation of child abuse is abbreviated as CHAB, and the other eight are abbreviated by using their first four letters.

^4^See footnote 1.
