# Supplementary material for: Justifications of emotional responses to eliciting situations: A narratological approach to the CAD hypothesis
Source: Front Psychol. 2022 Dec 6;13:1019485. doi: 10.3389/fpsyg.2022.1019485 (PMC9763725; doi:10.3389/fpsyg.2022.1019485)
Supplement: Supplementary file 1 [file Data_Sheet_1.docx]

**Appendices**

**Appendix 1a Categories of responses**

| Responses Situations | | | Community | | | Autonomy | | | Divinity | | | SUM |
| --- | --- | --- | --- | --- | --- | --- | --- | --- | --- | --- | --- | --- |
| Category | Sub-category | Samples | EMBE | DISR | BETR | HARM | CHEA | OPPR | CHAB | INCE | PATH |  |
| -des | -des;L | a bit worried (*yǒuxiē dānxīn*) | 0 | 0 | 0 | 1 | 0 | 0 | 0 | 0 | 0 | 1 |
|  | -des;M | worried (*dānxīn*) | 1 | 0 | 1 | 2 | 1 | 3 | 1 | 0 | 3 | 12 |
|  | -des;H | afraid (*hàipà*) | 0 | 0 | 1 | 2 | 0 | 0 | 0 | 0 | 2 | 5 |
| -hap | -hap \| o -norm: sympathy;L | poor (*kělián*) | 0 | 0 | 0 | 0 | 0 | 1 | 1 | 0 | 0 | 2 |
|  | -hap \| o -norm: sympathy;M | sympathic (*tóngqíng*) | 0 | 3 | 1 | 6 | 1 | 3 | 9 | 4 | 0 | 27 |
|  | -hap \| s -cap: helpless; L | Even the best judge becomes hopeless at family disputes (*qīngguān nánduàn jiāwùshì*) | 0 | 0 | 0 | 1 | 0 | 0 | 0 | 0 | 0 | 1 |
|  | -hap \| s -cap: helpless; M | helpless (*wúnài*) | 2 | 1 | 2 | 0 | 2 | 3 | 0 | 0 | 1 | 11 |
|  | -hap: antipathy: disgust; L | off-putting (*dǐchù*) | 0 | 2 | 0 | 0 | 0 | 0 | 0 | 1 | 2 | 5 |
|  | -hap: antipathy: disgust; M | disgust (*ěxin*) | 4 | 6 | 5 | 4 | 5 | 4 | 2 | 23 | 15 | 68 |
|  | -hap: antipathy: disgust;H | abominable (*zēngwù*) | 9 | 3 | 5 | 11 | 9 | 2 | 10 | 5 | 16 | 70 |
|  | -hap: antipathy: hate;L | resentful (*kěhèn*) | 0 | 0 | 0 | 1 | 0 | 0 | 1 | 0 | 0 | 2 |
|  | -hap: antipathy: hate;M | lament (*yuànhèn*) | 0 | 0 | 0 | 0 | 1 | 0 | 0 | 0 | 0 | 1 |
|  | -hap: antipathy: hate;H | loathe (*zēnghèn*) | 2 | 0 | 7 | 0 | 3 | 0 | 4 | 0 | 1 | 17 |
|  | -hap: misery;L | uncheerful (*bù kāixīn*) | 0 | 1 | 0 | 0 | 0 | 0 | 0 | 0 | 1 | 2 |
|  | -hap: misery;M | sad (*shāngxīn*) | 2 | 1 | 1 | 4 | 0 | 2 | 3 | 0 | 0 | 13 |
|  | -hap: misery;H | woeful (*bēiāi*) | 0 | 1 | 1 | 2 | 1 | 3 | 5 | 4 | 2 | 19 |
| -sat | -sat \| o -cap: contempt;L | look down on … (*kànbuqǐ*) | 0 | 0 | 0 | 0 | 3 | 1 | 0 | 1 | 0 | 5 |
|  | -sat \| o -cap: contempt;M | contempt (*bǐshì*) | 4 | 7 | 5 | 2 | 11 | 2 | 0 | 2 | 6 | 39 |
|  | -sat \| o -cap: contempt;H | spit (*tuòqì*) | 2 | 1 | 4 | 0 | 1 | 0 | 1 | 0 | 0 | 9 |
|  | -sat \| o -prop: shameful;L | be too shy to speak out (*xiūyúqǐchǐ*) | 0 | 0 | 0 | 0 | 0 | 0 | 0 | 1 | 0 | 1 |
|  | -sat \| o -prop: shameful;M | shame (*chǐrǔ*) | 2 | 0 | 5 | 0 | 0 | 0 | 0 | 1 | 2 | 10 |
|  | -sat \| s -prop: regret;L | pitiful (*yíhàn*) | 1 | 0 | 0 | 0 | 0 | 0 | 0 | 0 | 0 | 1 |
|  | -sat \| s -prop: regret;M | self-blame (*zìzé*) | 0 | 1 | 0 | 0 | 0 | 0 | 0 | 0 | 0 | 1 |
|  | -sat \| s -prop: regret;H | remorse (*cánkuì*) | 0 | 0 | 0 | 0 | 0 | 0 | 1 | 0 | 0 | 1 |
|  | -sat: displeasure: anger;L | untolerable (*bùkě rěnshòu*) | 3 | 1 | 0 | 1 | 1 | 7 | 0 | 0 | 0 | 13 |
|  | -sat: displeasure: anger;M | anger (*shēngqì*) | 7 | 3 | 5 | 3 | 6 | 5 | 5 | 0 | 6 | 40 |
|  | -sat: displeasure: anger;H | furious (*fènnù*) | 22 | 0 | 27 | 24 | 6 | 10 | 29 | 0 | 3 | 121 |
|  | -sat: displeasure: dissatisfied;L | hardly acceptable (*nányǐjiēshòu*) | 1 | 2 | 0 | 1 | 0 | 1 | 0 | 2 | 0 | 7 |
|  | -sat: displeasure: dissatisfied;M | dissatisfied (*bùmǎn*) | 0 | 1 | 0 | 2 | 3 | 2 | 0 | 0 | 1 | 9 |
|  | -sat: displeasure: dissatisfied;H | censure (*qiǎnzé*) | 1 | 1 | 0 | 1 | 1 | 5 | 1 | 0 | 0 | 10 |
|  | -sat: displeasure: upset;M | upset (*shīwàng*) | 2 | 2 | 0 | 1 | 3 | 0 | 0 | 1 | 0 | 9 |
|  | -sat: displeasure: upset;H | hopeless (*juéwàng*) | 0 | 0 | 0 | 1 | 2 | 0 | 0 | 0 | 0 | 3 |
|  | -sat: ennui;L | hit or miss (*wúsuǒwèi*) | 1 | 0 | 0 | 0 | 0 | 1 | 0 | 2 | 0 | 4 |
|  | -sat: ennui;M | flat (*mòrán*) | 0 | 0 | 0 | 0 | 0 | 0 | 0 | 0 | 1 | 1 |
|  | -sat: ennui;H | dumb (*mámù*) | 2 | 1 | 0 | 0 | 1 | 1 | 1 | 1 | 1 | 8 |
| -sec | -sec: n trust;M | distrust (*bùxìnrèn*) | 0 | 0 | 1 | 0 | 1 | 0 | 0 | 0 | 0 | 2 |
|  | -sec: disquiet: awkward;M | awkward (*gāngà*) | 0 | 0 | 0 | 0 | 0 | 0 | 0 | 2 | 1 | 3 |
|  | -sec: disquiet: disturbed; L | uneasy (*bùān*) | 0 | 1 | 1 | 0 | 1 | 3 | 0 | 1 | 1 | 8 |
|  | -sec: disquiet: disturbed; M | bored (*fán*) | 0 | 0 | 0 | 1 | 0 | 0 | 0 | 1 | 0 | 2 |
|  | -sec: surprise: startled;M | agitated (*jīnghuāng*) | 0 | 0 | 0 | 0 | 0 | 0 | 0 | 0 | 1 | 1 |
|  | -sec: surprise: startled;H | startled (*kǒngjù*) | 0 | 0 | 1 | 1 | 0 | 1 | 2 | 0 | 3 | 8 |
|  | -sec: surprise: surprise;M | surprise (*chījīng*) | 0 | 0 | 0 | 1 | 1 | 3 | 1 | 8 | 0 | 14 |
|  | -sec: surprise: surprise;H | shock (*zhènjīng*) | 0 | 1 | 2 | 5 | 2 | 4 | 5 | 7 | 0 | 26 |
| -under | -under;L | strange (*qíguài*) | 0 | 0 | 0 | 0 | 1 | 1 | 0 | 4 | 0 | 6 |
|  | -under;M | baffle (*yíhuò*) | 0 | 3 | 1 | 1 | 0 | 2 | 1 | 4 | 1 | 13 |
|  | -under;H | rediculous *(kěxiào)* | 1 | 0 | 0 | 0 | 0 | 2 | 0 | 2 | 0 | 5 |
| +under | +under; L | acceptable (*kějiēshòu*) | 0 | 4 | 1 | 1 | 2 | 3 | 0 | 0 | 1 | 12 |
|  | +under; M | understand (*lǐjiě*) | 0 | 5 | 0 | 0 | 1 | 0 | 0 | 0 | 0 | 6 |
|  | +under; H | totally fine (*wúkěhòufēi*) | 0 | 2 | 0 | 0 | 0 | 0 | 0 | 0 | 0 | 2 |
| neutral | neutral: quiet | quiet (*píngjìng*) | 2 | 11 | 0 | 1 | 3 | 1 | 0 | 4 | 3 | 25 |
|  | neutral: ordinary | normal *(zhèngcháng)* | 1 | 4 | 0 | 0 | 1 | 0 | 0 | 0 | 1 | 7 |
| +sat | +sat: interest;L | interested (*yǒuyìsi*) | 0 | 0 | 0 | 0 | 0 | 0 | 0 | 1 | 0 | 1 |
|  | +sat: interest;M | curious (*hàoqí*) | 0 | 0 | 0 | 1 | 0 | 0 | 0 | 1 | 0 | 2 |
| depend | | it depends (*kān qíngkuàng*) | 0 | 3 | 0 | 1 | 0 | 0 | 0 | 0 | 1 | 5 |
| mixed | | Mixed emotions (*qíngxù fùzá*) | 0 | 0 | 0 | 0 | 0 | 0 | 0 | 1 | 0 | 1 |
| SUM | | | 72 | 72 | 77 | 83 | 74 | 76 | 83 | 84 | 76 | 697 |

**Appendix 1a Responses to each situation**

**Figure 1** *Emotional responses to Embezzlement*

**Figure 2** *Emotional responses to Disrespect*

**Figure 3** *Emotional responses to Betrayal*

**Figure 4** *Emotional responses to Harm*

**Figure 5** *Emotional responses to Cheating*

**Figure 6** *Emotional responses to Oppression*

**Figure 7** *Emotional responses to Child abuse*

**Figure 8** *Emotional responses to Incest*

**Figure 9** *Emotional responses to Pathogen*

**Appendix 2 A summary of frequencies of responses’ categories**

|  | | Frequency | Percent | Cumulative Percent |
| --- | --- | --- | --- | --- |
| Valid | +sat: interest | 3 | .4 | .4 |
|  | +under | 20 | 2.9 | 3.4 |
|  | depend | 5 | .7 | 4.1 |
|  | mixed | 1 | .1 | 4.2 |
|  | neutral: quiet | 25 | 3.6 | 7.9 |
|  | neutral: ordinary | 7 | 1.0 | 8.9 |
|  | -under | 24 | 3.5 | 12.4 |
|  | -des | 18 | 2.6 | 15.0 |
|  | -hap\|o n_norm: sympathy | 26 | 3.8 | 18.8 |
|  | -hap\|s n_cap: helpless | 12 | 1.7 | 20.6 |
|  | -hap: antipathy: disgust | 142 | 20.7 | 41.3 |
|  | -hap: antipathy: hate | 20 | 2.9 | 44.2 |
|  | -hap: misery | 34 | 5.0 | 49.1 |
|  | -sat\|o -JUDG: contempt | 53 | 7.7 | 56.9 |
|  | -sat\|o n_prop: shameful | 11 | 1.6 | 58.5 |
|  | -sat\|s n_prop: regret | 3 | .4 | 58.9 |
|  | -sat: displeasure: anger | 170 | 24.8 | 83.7 |
|  | -sat: displeasure: dissatisfied | 24 | 3.5 | 87.2 |
|  | -sat: displeasure: upset | 12 | 1.7 | 88.9 |
|  | -sat: ennui | 13 | 1.9 | 90.8 |
|  | -sec: neg trust | 2 | .3 | 91.1 |
|  | -sec: surprise: startled | 8 | 1.2 | 92.3 |
|  | -sec: surprise: surprise | 40 | 5.8 | 98.1 |
|  | -sec: disquiet: awkward | 3 | .4 | 98.5 |
|  | -sec: disquiet: disturbed | 10 | 1.5 | 100.0 |
|  | Total | 686 | 100.0 |  |

**Appendix 3a Frequencies of narratorial categories**

| **Categories** | **Number** | **Percent** |
| --- | --- | --- |
| hetero_u | 768 | 92.60% |
| hetero_m | 8 | 1.00% |
| homo | 53 | 6.40% |
| Total | 829 | 100.00% |

**Appendix 3b Frequencies of different types of characters in total**

| **Categories** | **Number** | **Percent** |
| --- | --- | --- |
| equal IP-IV | 1 | 0.10% |
| imp IP-IV | 53 | 6.40% |
| imp IP-CV | 9 | 1.10% |
| imp IP | 95 | 11.50% |
| imp CP | 7 | 0.80% |
| imp IV | 32 | 3.90% |
| imp CV | 21 | 2.50% |
| int IP-IV | 4 | 0.50% |
| int IP-CV | 2 | 0.20% |
| int CP-CV | 1 | 0.10% |
| int IP | 2 | 0.20% |
| int CP | 2 | 0.20% |
| int IV | 4 | 0.50% |
| int pre IP-sub IV | 2 | 0.20% |
| int sub CV | 6 | 0.70% |
| pre IP-sub IV | 34 | 4.10% |
| pre IP-sub CV | 12 | 1.40% |
| pre CP-sub IV | 1 | 0.10% |
| pre CP-sub CV | 1 | 0.10% |
| pre IP | 59 | 7.10% |
| pre CP | 10 | 1.20% |
| sub IV | 25 | 3.00% |
| sub CV | 24 | 2.90% |
| social IP-IV | 4 | 0.50% |
| social IP-CV | 37 | 4.50% |
| social CP-IV | 1 | 0.10% |
| social CP-CV | 1 | 0.10% |
| social IP | 1 | 0.10% |
| social CP | 4 | 0.50% |
| social IV | 3 | 0.40% |
| social CV | 3 | 0.40% |
| N | 347 | 41.90% |
| nar IV | 13 | 1.60% |
| nar CV | 1 | 0.10% |
| nar other | 5 | 0.60% |
| Other | 2 | 0.20% |
| Total | 829 | 100.00% |

**Appendix 3b’ Combined categories of participants related to the three major emotions**

| Imp_V | Imp IC-IV | 62 |
| --- | --- | --- |
|  | Imp IC-CV |  |
|  | Imp IV |  |
|  | Imp CV |  |
| Imp_P | Imp IP | 34 |
|  | Imp CP |  |
| Sub_V | pre IP-sub IV | 31 |
|  | pre IP-sub CV |  |
|  | pre CP-sub IV |  |
|  | pre CP-sub CV |  |
|  | sub IV |  |
|  | sub CV |  |
| Pre_P | pre IP | 37 |
|  | pre CP |  |
| Social_V | social IP-IV | 42 |
|  | social CP-IV |  |
|  | social IP-CV |  |
|  | social CP-CV |  |
|  | social IV |  |
|  | social CV |  |
|  | Int IP-IV |  |
|  | Int IP-CV |  |
|  | Int CP-CV |  |
|  | Int IP |  |
|  | Int CP |  |
|  | Int IV |  |
|  | Int pre IP-sub IV |  |
|  | Int sub CV |  |
|  | nar IV |  |
|  | nar CV |  |
| N | N | 97 |
| Total | 303 | |

**Appendix 3c Frequencies of categories of basis**

| **Categories** | | | **Number** | **Percent** |
| --- | --- | --- | --- | --- |
| reaction | | n +hap | 2 | 0.24% |
|  |  | N | 56 | 6.76% |
| Interpretation | social esteem | JUDG: +norm | 17 | 2.05% |
|  |  | JUDG: -norm | 27 | 3.26% |
|  |  | JUDG: -cap | 21 | 2.53% |
|  |  | JUDG: -ten | 9 | 1.09% |
|  |  | JUDG: n -ver | 1 | 0.12% |
|  |  | JUDG: -ver | 33 | 3.98% |
|  | social sanction | JUDG: n -prop | 1 | 0.12% |
|  |  | JUDG: +prop_auto | 3 | 0.36% |
|  |  | JUDG: -prop_auto | 130 | 15.68% |
|  |  | JUDG: -prop_com | 86 | 10.37% |
|  |  | JUDG: -prop_div | 141 | 17.01% |
|  | jurisdiction & others | JUDG: -law | 30 | 3.62% |
|  |  | JUDG: neutral | 1 | 0.12% |
| comment | appreciation | APPR: +reac | 2 | 0.24% |
|  |  | APPR: +comp | 3 | 0.36% |
|  |  | APPR: -comp | 5 | 0.60% |
|  |  | APPR: +val | 7 | 0.84% |
|  |  | APPR: -val | 2 | 0.24% |
|  | accountability | +acc | 57 | 6.88% |
|  |  | -acc | 33 | 3.98% |
| narrative completion | consequence | n -auto | 6 | 0.72% |
|  |  | -auto | 119 | 14.35% |
|  |  | -com | 12 | 1.45% |
|  |  | -div | 9 | 1.09% |
|  | solubility | +sol | 5 | 0.60% |
|  |  | -sol | 11 | 1.33% |
| Total | | | 829 | 100% |

**Appendix 3c’ Frequencies of combined categories of basis**

| Combined categories | Original categories | Number | Percent |
| --- | --- | --- | --- |
| APPR_neg | APPR: -comp | 9 | 1.1% |
|  | APPR: -val |  |  |
| APPR_pos | APPR: +reac | 12 | 1.4% |
|  | APPR: +comp |  |  |
|  | APPR: +val |  |  |
| JUDG: -norm | JUDG: -norm | 27 | 3.3% |
| JUDG: -cap-ten | JUDG: -ten | 30 | 3.6% |
|  | JUDG: -ver |  |  |
| JUDG: -ver | JUDG: -ver | 33 | 4.0% |
| JUDG: -prop_auto | JUDG: -prop_auto | 249 | 30.0% |
|  | -auto |  |  |
| JUDG: -prop_com | JUDG: -prop_com | 98 | 11.8% |
|  | -com |  |  |
| JUDG: -prop_div | JUDG: -prop_div | 150 | 18.1% |
|  | -div |  |  |
| JUDG: -law | JUDG: -law | 30 | 3.6% |
| JUDG_n neg | JUDG: n -ver | 29 | 3.5% |
|  | JUDG: +auto |  |  |
|  | JUDG: n -prop, |  |  |
|  | JUDG: neutral |  |  |
|  | n -auto |  |  |
| -acc | -acc | 33 | 4.0% |
| +acc | +acc | 57 | 6.9% |
| -sol | -sol | 11 | 1.3% |
| +sol | +sol | 5 | .6% |
| N | N | 56 | 6.8% |
| Total |  | 829 | 100.0% |
